# Bilateral endogenous fungal endophthalmitis: A case report

**DOI:** 10.1097/MD.0000000000033585

**Published:** 2023-04-21

**Authors:** Hao Wang, Yongye Chang, Yifan Zhang, Rong Yang, Huijun Shi, Minglian Zhang

**Affiliations:** a Department of TCM Ophthalmology, Hebei Eye Hospital, Hebei province, China; b Hebei Provincial Key Laboratory of Ophthalmology, Hebei province, China; c Hebei Provincial Clinical Research Center for Eye Diseases, Hebei province, China; d School of Life Sciences and Food Engineering, Hebei University of Engineering, Hebei province, China.

**Keywords:** antifungal medication, diagnosis, endogenous fungal endophthalmitis, vitrectomy

## Abstract

Endogenous fungal endophthalmitis is a challenging condition. There are no universally accepted diagnostic or management protocols. We share a case of endogenous fungal endophthalmitis who was successfully treated, focusing on the diagnostic and treatment procedures.

**Patient concerns:**

A 31-year-old female with a history of fungal vaginitis and tinea corporis presented with progressive visual decrease in both eyes after having an induced abortion. Her best corrected visual acuity at presentation was 20/1000 in her right eye and 20/250 in her left eye. Upon slit lamp examination, mild inflammatory reaction in the anterior chamber was found. Dilated fundus examination revealed a hazy view of the optic disc and posterior retina, and there was a whitish mass with “string and pearls” just in front of the macular region in each eye.

**Diagnoses:**

Bilateral fungal endogenous endophthalmitis was diagnosed empirically, which was confirmed later by deoxyribonucleic acid sequencing and culture of intraocular fluid.

**Interventions:**

Oral itraconazole and intravitreal voriconazole were administered to the patient at first. The intraocular inflammation was partially responsive to the medication, yet the visual acuity persisted to deteriorate and the vitreous whitish masses became more prominent. Then vitrectomy procedures were carried out and oral itraconazle was switched to intravenous fluconazole. The antifungal treatment lasted for 8 weeks.

**Outcomes:**

The intraocular inflammation alleviated and visual acuity improved after vitrectomy. At the 9-month follow-up visit, the patient’s best corrected visual acuity was 20/40 in the right eye and 20/30 in the left eye. There was no intraocular inflammatary reaction, and retinal scar was noticed in each eye.

**Lesson:**

Early and correct diagnosis, coupled with prompt and aggressive treatment, is crucial for cases of fungal endogenous endophthalmitis. Deoxyribonucleic acid sequencing techniques can contribute to early diagnosis, while vitrectomy may be necessary when antifungal medication is insufficient in controlling the condition.

## 1. Introduction

Endogenous fungal endophthalmitis is an uncommon condition, typically presenting with nonspecific symptoms, which are indistinguishable from those of chronic uveitis. As there is a lack of systematic studies and randomized clinical trials, no universally accepted diagnostic or management protocols for this condition were available currently. Herein, we present a case of bilateral endogenous fungal endophthalmitis who was successfully treated with antifungal medication and vitrectomy.

## 2. Case presentation

**Figure 1.**
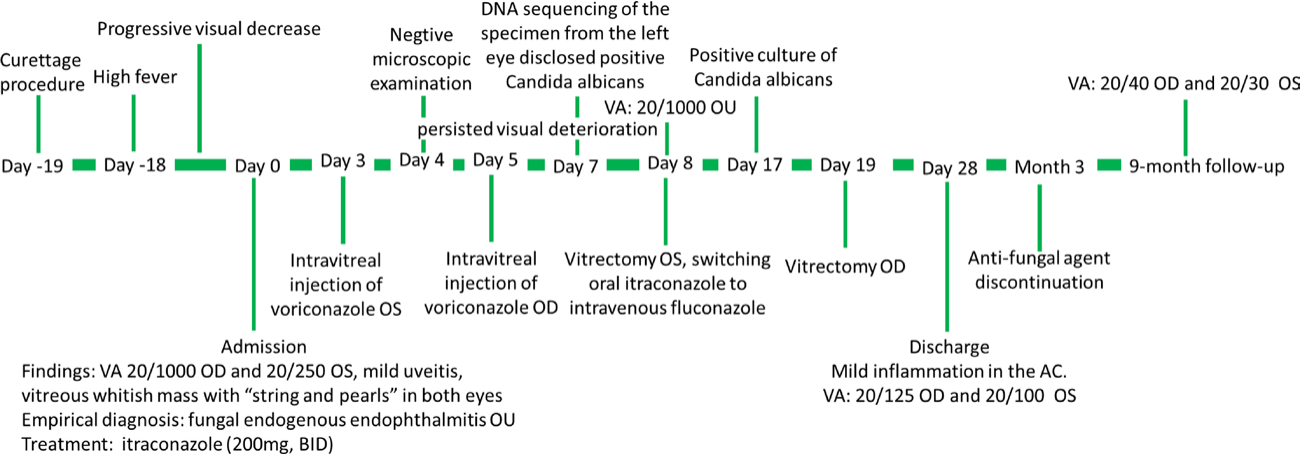
Clinical timeline.

A 30-year-old Chinese women, who had a curettage procedure to terminate a pregnancy at 8 weeks of gestation, developed high fever 1 day after the induced abortion. She was treated with systemic antibiotics and nonsteroidal antipyretic drugs for 1 week, after which the body temperature returned to normal, but decrease in vision in both eyes was noted. As the visual impairment progressed, there was no eye redness, pain, irritation, tearing or other accompanying symptoms. The patient was referred to Hebei eye hospital about 2 weeks after the onset of visual symptom (Fig.1). Her medical history was remarkable for fungal vaginitis and tinea corporis. She lived in the rural region, and did not use tobacco, alcohol, or any other illicit drugs. She had had 3 pregnancies (2 cesarean births, 1 induced abortion). Her family had no history of prolonged febrile illnesses, infectious diseases, arthritis, autoimmune diseases, or malignancy.

**Figure 2.**
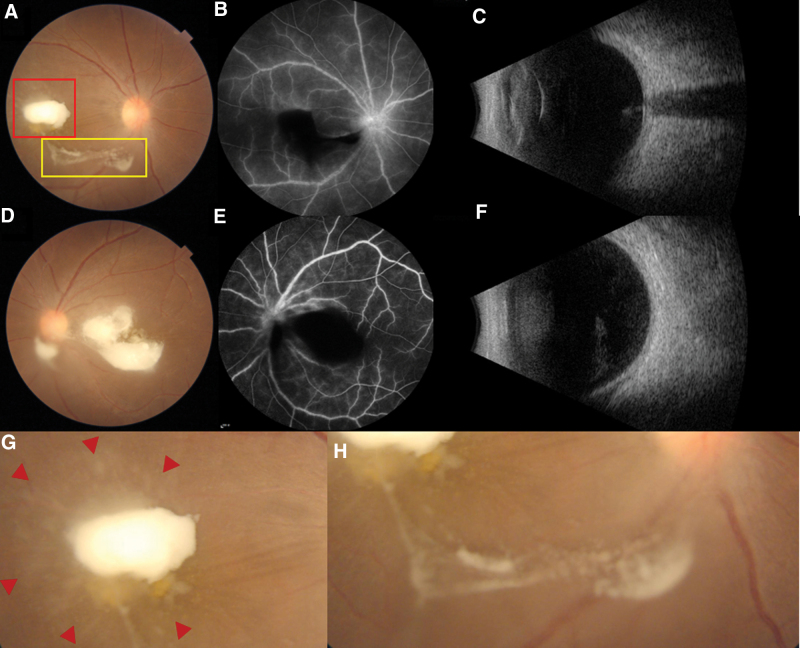
Upon admission fundus examination showed a hazy view of optic disc and posterior retina. There was a whitish mass (Fig. 2A and Fig. 2D) with “string and pearls” (yellow box in Fig. 2A and Fig. 2H) in each eye. The mass in the right eye emit umbrella-like filaments attached to the retina (red box in Fig. 2A and Fig. 2G). FFA showed optic disc and posterior retinal vessels leakage (Fig. 2B and Fig. 2E). Ultrasonic B scan demonstrated dense punctate echoes in the posterior vitreous and flat retina in both eyes (Fig. 2C and Fig. 2F). FFA = fundus fluorescein angiography.

On her initial presentation to our hospital, best corrected visual acuity (BCVA) was 20/1000 in her right eye and 20/250 in her left eye. Intraocular pressure was within normal limits. Slit lamp examination revealed no conjunctival hyperemia or keratic precipitates on the inferior part of the cornea; however, there were 2 + cells and 1 + flare in the anterior chamber of both eyes. The irises were normal, with no visible nodules or neovascularization. Dilated fundus examination showed a hazy view of the optic disc and posterior retina, and there was a whitish mass with “string and pearls” just in front of the macular region in each eye. The mass in the right eye was smaller, emitting umbrella-like filaments attached to the retina (Fig.2).

**Figure 3.**
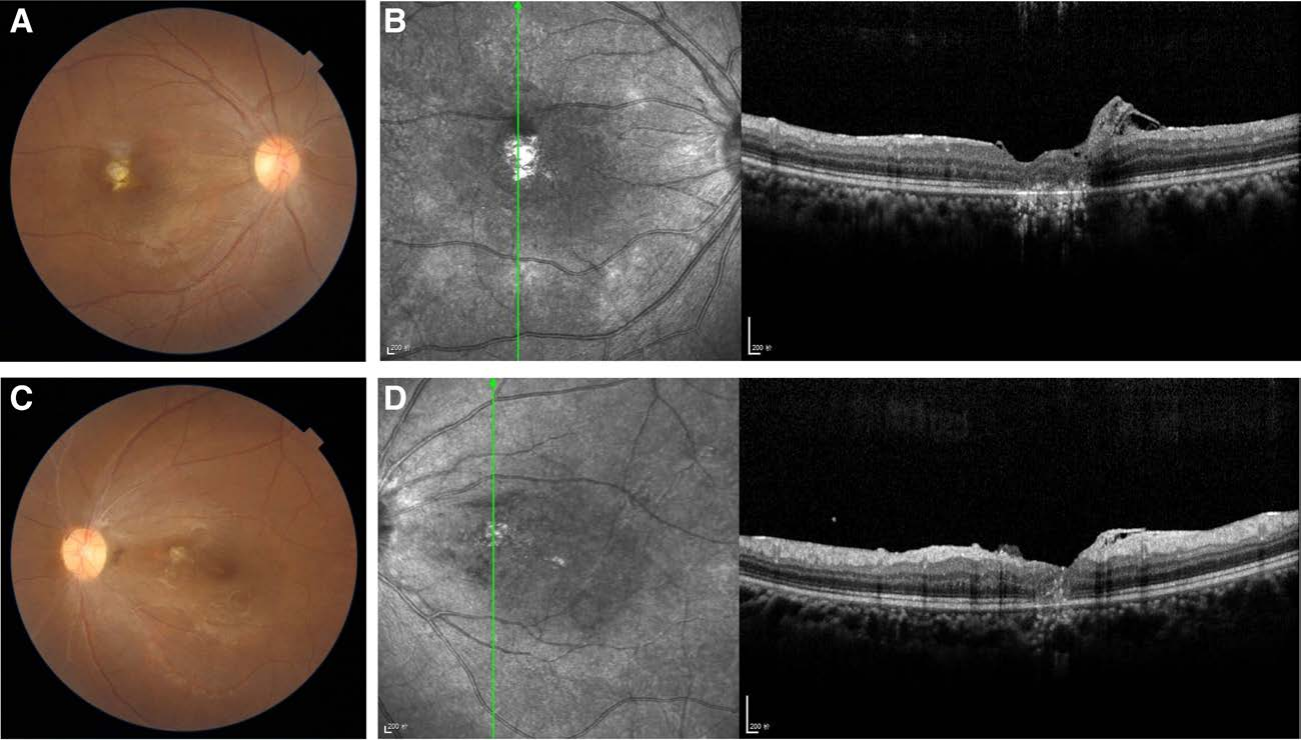
At the 9-month follow-up, yellow whitish retinal scars were noticed in both eyes (Fig. 3A, Fig. 3C). OCT showed focal disorganized leisure around the fovea (Fig. 3B, Fig. 3D). OCT = optic coherene tomography.

The patient was admitted. Laboratory examination showed mild anemia (RBC 3.66 × 1012/L, hemoglobin 95 g/L) and erythrocyte sedimentation rate was 28 mm/hour. Ultrasonic B scan demonstrated dense punctate echoes in the posterior vitreous and flat retina in both eyes (Fig. 2). Fundus fluorescein angiography showed optic disc and posterior retinal vessels leakage (Fig. 2). Liver and renal function tests, C reactive protein, syphilis, human immunodeficiency virus, urine analysis, and X-ray of the chest all came to negative. Bilateral fungal endogenous endophthalmitis was diagnosed empirically. Oral itraconazole (200 mg, BID) was administered, as well as topical treatment with 1 % tropicamide eyedrops. Intravitreal injection of voriconazole (100 ug/0.1 mL) was administered in the left eye on hospital day 3 and in the right eye on hospital day 5. Vitreous fluid was collected during the procesure and sent for microscopic examination, culture, and metagenomic next generation sequencing. The microscopic examinations were negative but the next generation sequencing of the specimen from the left eye disclosed positive Candida albicans 4 days after the intravitreal injection. The intraocular inflammation was partially responsive to the medication, yet the visual acuity persisted to deteriorate and the vitreous whitish masses became more prominent and thicker. Then vitrectomy was performmed in the left eye on hospital day 8, and oral itraconazole was switched to intravenous fluconazole (200 mg/day). After the surgery, the inflammation in the left eye alleviated. Culture of the specimen obtained from the intravitreal injection was negative, but that of the specimen from vitrectomy comfirmed the presence of Candida albicans on hospital day 17. On hospital day 19 the right eye was also vitrectomized. On hospital day 28, the patient was discharged with mild inflammation in the anterior chamber in both eyes. Her BCVA improved to 20/125 in the right eye and 20/100 in the left eye. The antifungal treatment lasted for 8 weeks. At the 9-month follow-up, there was no intraocular inflammatary reaction, and retinal scar was noticed in each eye (Fig.3). Her BCVA improved to 20/40 in the right eye and 20/30 in the left eye.

## 3. Discussion

Endophthalmitis is a rare and severe intraocular infection caused by bacteria or fungus, which can be vision-threatening and results in devastating visual impairment. It is classified into exogenous and endogenous subtypes. Exogenous endophthalmitis is typically related to trauma or surgery, while endogenous endophthalmitis is usually a result of hematogenous dissemination of pathogens from another site of coexisting infection. According to the literature, endogenous endophthalmitis made up approximately 5.0% to 10% of all endophthalmitis case.^[1–3]^ Of the endogenous cases, approximately 20% to 60% were caused by fungi,^[3–5]^ with Candida albicans and Aspergillus flavus being the most common pathogens, together accounting for about 50% to 70% of fungal endophthalmitis.^[3,6,7]^

In most cases, endogenous fungal endophthalmitis is the result of immunosuppression coinciding with fungemia. Immunosuppression alone is not sufficient for fungal endophthalmitis, as there are many predisposing factors including intravenous antibiotic, major surgery, intravenous catheters, intravenous infusions, steroid or immunosuppressant treatment, and intravenous drug abuse.^[7]^ Fungal dissemination is particularly frequent in diabetic patients, neonates, and burn patients.^[8]^ Pregnancy could also be a risk factor for fungal infection. Culture rates of Candida species from the vagina increase twofold during pregnancy due to hormonal changes that predispose women to fungal colonization during pregnancy.^[9]^ Therefore, it is possible that this patient had a transient Candida invasion to the bloodstream during the induced abortion procedure that might have resulted in high fever, Candida chorioretinal seeding and vitreous spreading. Additionally, She also had anemia, which could also be a risk factor for endophthalmitis.^[10–13]^ Anemia may compromise the immune system and increase the risk of opportunistic infection.

Endogenous fungal endophthalmitis is usually subacute. In this case, it was more than 2 weeks between the ocular fungal infection onset and the admittance. Pulimood et al^[14]^ reported the mean latent period of this condition could be as long as 30 days. Only 3% of those invoved in endogenous fungal endophthalmitis presented within 1 week. Slowly progressing inflammation should raise the suspicion for fungal endophthalmitis,^[15]^ since the fungi usually proliferate much more slowly than the bacteria. Bilateral eye involvement was more prevalent in endogenous fungal endophthalmitis than in bacterial endophthalmitis.^[4]^

The clinical manifetistation of fungal endophthalmitis is variable. Peripheral or early fungal lesions may be asymptomatic. In most cases, the patients present with decreased vision, floaters, ocular pain, photophobia, eye redness, anterior chamber cells, flare or hypopyon, vitreous inflammation and chorioretinitis. Severe vitreous inflammation obscures the view of the fundus. Different species of fungi can lead to different clinical changes. Aspergillus-mediated endophthalmitis is usually more severe than Candida endophthalmitis, as Aspergillus can grow in subretinal space, invade the retinal and choroidal vessels and the choriocapillaris, resulting in extensive necrosis of the choroid, retinal pigment epithelium, and retina. Candida, on the other hand, seeds in the choroid and then spreads into the retina and vitreous cavity, resulting in the appearance of intravitreal “puff ball” abscesses without intensive infiltration in the retina.^[16]^ Candida endophthalmitis typically presents with multiple creamy white or fluffy, well-circumscribed retinal and vitreous lesions connected by strands, forming a “string of pearls” appearance. The hallmark for the diagnosis of Candida infection is the presence of a fluffy creamy white lesion at the level of the retina and choroid that is usually associated with vitreous haze.^[17]^

Clinical symptoms and signs are usually inadequate for the diagnosis of fungal infection, which should be supported by the microbiological examination. Methods for microbiological detection include direct microscopic examination, culture, polymerase chain reaction and deoxyribonucleic acid (DNA) sequencing. Microscopical examination and culture of the vitreous or aqueous humor are the most widely used methods to search for the pathogen, while culture can also provide the drug susceptibility testing. However, the results of microscopy or culture are often affected by the vitreous sampling technique and prior antimicrobial use. It is reported that the culture positivity is just 25.6% to 44.1%.^[18–22]^ Additionally, culture may take 1 to 2 weeks to yield a positive result, resulting in a delay in receiving proper treatment. In this case, the method of metagenomic next generation sequencing was applied, which took just 4 days to accurately identify Candida infection, whereas the traditional method of culture either yielded a negative result, or exceeded 1 week to confirm the fungal presence. DNA sequencing or polymerase chain reaction (PCR) techniques, on 1 hand, can detect DNA from live or dead organisms, significantly increasing the yield of detection. On the other hand, they substantially reduce the time for etiological diagnosis. Anand et al^[23]^ studied 43 intraocular specimens of fungal endophthalmitis cases and found culture positivity in 24 (55.8%) cases compared to PCR positivity in 32 (74.4%). Sowmya and colleagues reported the PCR positivity of infectious endophthalmitis as high as 100%, compared to culture’s 37.5%.^[21]^ DNA sequencing does not target specific species. Zhu and colleagues reported the positive rates of metagenomic next generation sequencing and culture were 88.89% (32/36) and 27.78% (10/36) respectively.^[22]^ Dhanshree and colleages reported the sensitivity and specificity of next generation sequencing was as high as 87.5% and 100% respectively.^[20]^ However, neither of these molecular biological methods can provide information on drug susceptibility. Thus, the combination of the culture and morden molecular biological methods should be the preferred choice. Clinicians must balance the clinical and laboratory data while observing the response to the treatment.

Standard treatment protocols for fungal endophthalmitis are not available due to its rarity. The treatment mainstays are intravitreal or systemic antifungal medication and vitrectomy surgery.

Intravitreal injection of antifungal agents is a crucial method to attain a high intraocular level of the drug while limiting drug-related systemic toxicity. Raised fungal lesions on the surface of the retina or protruding into the vitreous necessitate intravitreal injection. Currently, only amphotericin B (5 to 10 ug/100 uL)^[24]^ and voriconazole (100 ug/100 uL)^[25]^ are clinically used for intravitreal injection. The half-life of amphotericin B is 8.9 days in the vitreous of noninflamed phakic eyes and 1.8 days in the aphakic vitrectomized eye. While the half-life of voriconazole is 2.5 to 6.5 hours in vitreous of noninflamed phakic eyes and 2.5 hours in aphakic vitrectomized eyes.^[26]^ Due to the short half-life of these agents in the eye, frequent intravitreal injections are required to maintain therapeutic intraocular concentrations. Therefore, oral or intravenous administration of antifungal agents becomes necessary for convenience. Systemic use of antifungal agents is also essential for individuals with fungemia at presentation. Azole class drugs such as fluconazole and voriconazole are frequently administered systemically for fungal endophthalmitis,^[27,28]^ as they can penetrate into the eye and achieve high intraocular levels. Flucytosine, ketoconazole, itraconazole, micafungin, anidulafungin et al are also used systemically for fungal endophthalmitis.^[29]^

Although there are no established guidelines regarding the necessity and timing of pars plana vitrectomy in fungal endophthalmitis, early and complete vitrectomy is recommended by some researchers.^[30]^ Vitrectomy is typically both diagnostic and therapeutic. The removal of the vitreous reduces the fungal load, enhances the effect of the antifungal drugs and provides a specimen for laboratory test and culture. A recent review of several series of patients with endogenous fungal endophthalmitis found that vitrectomy was performed in 24.2% to 56.9% of eyes.^[31]^ In a retrospective cohort of 44 eyes with endogenous fungal endophthalmitis, Sallam et al^[18]^ found that early vitrectomy reduced the risk of retinal detachment significantly. In this case, vitrectomy accelerated the resolution of fungal infection and avoided complications sucn as retinal detachment.

## 4. Conclusion

Endogenous fungal endophthalmitis is a vision-threatening entity. Early and correct diagnosis, coupled with prompt and aggressive treatment, is crucial for the prognosis of fungal endogenous endophthalmitis.

## Acknowledgments

The authors would like to thank the patient and her family for providing informed consent for publication.

## Author contributions

**Conceptualization:** Minglian Zhang.

**Data curation:** Hao Wang, Yongye Chang.

**Funding acquisition:** Yongye Chang, Huijun Shi, Minglian Zhang.

**Investigation:** Hao Wang, Yongye Chang, Rong Yang, Huijun Shi, Minglian Zhang.

**Writing – original draft:** Hao Wang, Yongye Chang.

**Writing – review & editing:** Yifan Zhang, Minglian Zhang.
